# Light-Induced Degradation of Tamoxifen in Liquid Formulations: Multivariate Kinetic Profiling, Stabilization Strategies, and Estrogen Receptor Binding

**DOI:** 10.1021/acsomega.5c03822

**Published:** 2025-10-16

**Authors:** Maria Antonietta Occhiuzzi, Martina Chieffallo, Giuseppina Ioele, Giancarlo Di Pinto, Giuseppe Cirillo, Michele De Luca, Antonio Garofalo, Fedora Grande

**Affiliations:** † Department of Pharmacy, 18950 Health and Nutritional Sciences, University of Calabria, Rende 87036, Italy; ‡ Medical Oncology Unit, Ferrari Hospital, Castrovillari 87012, Italy

## Abstract

Tamoxifen is the most prescribed drug for the treatment of breast cancer in premenopausal women and prevention of tumor recurrence. The anticancer effect is attributed to its ability to modulate estrogen receptor activity, with the drug’s metabolites being more effective than the parent compound. Tamoxifen is sensitive to environmental conditions, leading to the formation of degradation products that may, however, retain biological activity. Herein, the photodegradation of tamoxifen in oral formulations was studied by combining spectrophotometric methodologies and multivariate analysis. The four photoproducts identified have been studied. Stabilization strategies were explored, evaluating both protective packaging precautions and the addition of chemical stabilizers, such as ascorbic acid and quercetin. Molecular docking simulations revealed that all four photoderivatives are capable of binding to the estrogen receptor, suggesting that these compounds may retain, or contribute to, the drug’s antitumor activity. These findings not only underscore the importance of formulation and storage conditions in preserving tamoxifen stability and therapeutic efficacy but also provide the first integrated multivariate kinetic and molecular docking analysis of its photodegradation in liquid formulations, offering novel insights into both stability and residual pharmacological activity.

## Graphical abstract

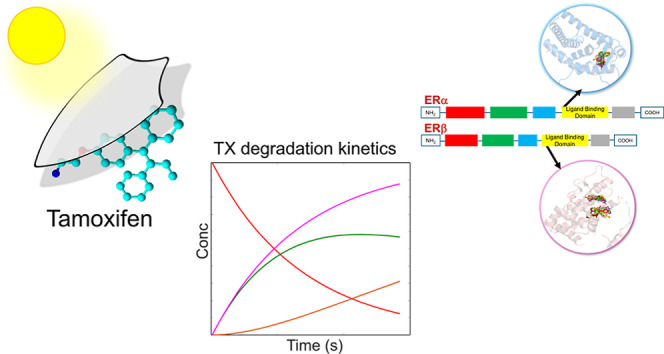

## 1 Introduction

Breast cancer is one of the most commonly diagnosed cancers and remains the leading cause of cancer-related deaths among women worldwide^1^ This type of cancer is classified into three subtypes based on the expression levels of estrogen receptor (ER), progesterone receptor (PR), or human epidermal growth factor 2 receptor (HER2) in cancer cells: hormone receptor-positive (ER+ and/or PR+), HER2 positive (HER2+), and triple-negative breast cancer (ER-, PR-, and HER2-). Approximately 60–75% of all diagnosed breast cancers are ER + for which endocrine therapy is the mainstay of systemic treatment.^2^ Earlier antihormonal strategies, such as ethamoxytriphetol, showed limited potency associated with a high risk of serious side effects on the CNS, precluding clinical application. This led to the development of safer and more effective agents such as tamoxifen (TX, (*Z*)-2-(4-(1,2-diphenylbut-1-en-1-yl)­phenoxy)-*N*,*N*-dimethylethan-1-amine)).^3,4^ This agent is widely used in premenopausal women and in adjuvant therapy after breast surgery, and it is also effective in chemoprevention in high-risk patients, reducing the rate of recurrence and mortality even several years after treatment.^5–7^ TX and its most active metabolites exert their effects by binding the ER, thus precluding estrogen transcriptional activity in breast tissues.^8^ However, this drug is endowed with partial agonist activity in the uterus, contributing to side effects such as an increased risk of endometrial carcinoma and uterine sarcoma. Additional challenges associated with TX treatment include the development of drug resistance, along with various side effects such as hot flashes and liver abnormalities.^9^ It is well-known that the activity of TX is only in part due to the drug itself, with some metabolites, particularly HTX and DHTX, even showing up to 30–100 times greater activity against ER-dependent human breast cancer cell proliferation (Supplementary, Figure S1).^10^ However, its marked sensitivity to metabolic transformations and chemical or physical stressors indicates a high overall reactivity. TX is known to undergo a rapid rearrangement upon light exposure, leading to products of uncertain biological relevance. Recent stability studies have provided a detailed photodegradation profile: the native drug initially undergoes partial isomerization to *E*-TX. Upon continued photoirradiation, both isomers are partially converted into their respective phenanthrene derivatives, D1 and D2. Additionally, in the presence of oxygen, both olefinic isomers are further transformed into a photooxidation ketone metabolite, D3, resulting in a mixture of five distinct products^11,12^ (Supporting Information Figure S1). TX and its byproducts have been widely investigated through various analytical methods, including chromatographic techniques with UV/vis, fluorescence, or mass spectrometry detection.^11–16^ Moreover, spectroscopic methods have been employed to analyze the drug in pharmaceutical formulations.^17^

TX is commonly formulated as tablets or solutions for oral administration. Tablets contain tamoxifen citrate with various excipients such as lactose, starch, magnesium stearate, and others to aid in the manufacturing process and to improve stability and bioavailability.^18^ Oral solutions, formulated for patients with swallowing difficulties, consist of tamoxifen citrate dissolved in suitable solvents along with flavoring agents to improve palatability.^19,20^ Since tamoxifen is susceptible to degradation upon exposure to light, a photostability investigation, especially for liquid formulation, could be useful to ensure efficacy and safety.

In the present research work, a commercially available liquid formulation of tamoxifen citrate was investigated to assess its photostability in accordance with ICH (International Council for Harmonization of Technical Requirements for Pharmaceuticals for Human Use) guidelines. To enhance drug stability under light exposure, multiple stabilization strategies were explored. These included both the use of an amber glass container as a physical light-shielding barrier and the incorporation of two antioxidant agents, ascorbic acid (ASA) and quercetin (QUE), both of which are well-known for their stabilizing properties in pharmaceutical and cosmetic formulations.^21^ Stability monitoring was conducted by UV/vis spectrophotometry, and the spectral data were processed by multivariate curve resolution-alternating least squares (MCR-ALS), a chemometric approach particularly effective for resolving the spectral and concentration profiles of complex systems undergoing transformation.^22^ An independent HPLC-DAD method was developed to validate the results obtained by the multivariate analysis.^13^ Finally, molecular docking simulations were carried out to assess whether the main photoproducts could retain affinity for the ligand-binding domains of ERα and ERβ, using the crystallographic pose of HTX as a reference, in order to evaluate their potential contribution to residual or altered biological activity. By combining photostability profiling, stabilization strategies, and receptor-binding simulations, our work introduces an innovative and comprehensive framework to evaluate the therapeutic relevance of drug degradation products.

## 2 Methods

### 2.1 Chemicals

The TX standard, with a certified purity >99%, was purchased from Merck (Milan, Italy) and used as received. All samples were prepared using distilled water and analytical-grade reagents. Ethanol, glycerol, propylene glycol, sorbitol, ascorbic acid, quercetin, ammonium acetate, liquorice and aniseed flavors, HPLC-grade water, and methanol were obtained from Merck (Milan, Italy).

### 2.2 Instruments

Photodegradation experiments were conducted in a SUNTEST Suntest CPS + light cabinet (Atlas, Milan, Italy), which was equipped with a xenon arc lamp, following the light irradiation conditions specified in the ID65 standard of the International Council for Harmonisation of Technical Requirements for Pharmaceuticals for Human Use (ICH).^23^ UV/vis spectra were recorded using an HP-Agilent 8453 UV/vis molecular absorption spectrophotometer (Agilent Technologies, CA, USA) with a diode array detector (DAD). HPLC analysis was carried out using an Agilent 1100 series chromatograph (Agilent Technologies, CA, USA) equipped with a binary pump delivery system and a diode array UV/vis detector. Spectrophotometer ChemStation software (Agilent Technologies, CA, USA) was employed for acquiring experimental data and converting raw UV spectra files (.sd) into human-readable files (.csv) suitable for direct importation into MATLAB R2023a (The MathWorks, Inc., MA, USA). All chemometric analyses were conducted within the MATLAB computing environment, utilizing the MCR-ALS procedure (GUI version 2.0).^24^

### 2.3 Experimental Procedures

Primary stock solutions of TX ASA and QUE (200 mg/L) were prepared by using ethanol as the solvent. The TX stock solution was also used to prepare the oral formulation by mixing it with the excipient mixture according to the recipe provided by Rosemont Pharmaceuticals Ltd. (Leeds, UK) for the commercial product named SOLTAMOX. The liquid oral formulation solvent (OFS) was prepared by mixing the following ingredients to obtain a final solution with a total volume of 50 mL: ethanol (7.50 g), glycerol (22.50 g), propylene glycol (5.03 g), sorbitol solution (noncrystallizing) (10.00 g), natural aniseed flavoring (0.005 g), liquorice flavoring (0.01 g), and water to reach a volume of 50 mL. All standard samples and formulations were diluted to a final TX concentration of 20 mg/L. The solutions were transferred into perfectly sealed quartz cuvettes for the main kinetic investigation. UV/vis spectra were recorded over the wavelength range of 220–450 nm, starting immediately after preparation (*t* = 0 min), then every 2 min for the first 30 min, and every 5 min thereafter until the end of a 4 h exposure. Photodegradation tests were conducted in accordance with the ICH Q1B (Option 2) guidelines using a SUNTEST CPS + solar simulator (Atlas, Milan, Italy) equipped with a xenon arc lamp. Irradiation parameters included a wavelength range of 300–800 nm, a power density of 400 W/m^2^ (24 kJ/(min·m^2^)), and a constant temperature of 25 °C.^23^ All photodegradation experiments were performed in triplicate. In addition to quartz cells, to simulate realistic packaging conditions, additional experiments were performed using neutral (clear) and amber borosilicate glass vials with a path length of 1 cm path length. The photoprotective effect of amber glass was evaluated by comparing the TX residual percentages with those obtained in quartz and clear glass cells. The stability of TX was also tested in the presence of substances with potential stabilizing effects.^25^ Binary mixtures (TX + ASA, TX + QUE) and the ternary mixture (TX + ASA + QUE) were prepared in both ethanol and OFS, with stabilizer concentrations ranging from 2.5 to 10 mg/L.^26,27^ The complete experimental design is summarized in Table 1. For independent confirmation of the degradation product, HPLC analysis was performed on a reverse-phase Luna LC column (250 × 4.60 mm, 5 μm, C18, Phenomenex, Torrance, CA) with a mobile phase consisting of methanol and ammonium acetate buffer (pH 4.5) in an 80:20 (v/v) ratio, prefiltered through a 0.45 μm filter. The injection volume was 20 μL, and the flow rate of the mobile phase was 1.0 mL/min at a room temperature of 20 °C. HPLC analysis was performed on TX solutions (1 mg/mL) exposed to light immediately after preparation (*t* = 0 min) and after 2 h.^13^

**Table 1:** Experimental Design and Sample Preparation

			sample composition		
data matrix	container^b^	solvent	TX (mg/L)	ASA (mg/L)	QUE (mg/L)
**D**_**TXe1–3**_ ^a^	Q	ethanol	20.0		
**D**_**TXo1–3**_	Q	OFS	20.0		
**D**_**ngTXe1–3**_	NG	ethanol	20.0		
**D**_**ngTXo1–3**_	NG	OFS	20.0		
**D**_**agTXe1–3**_	AG	ethanol	20.0		
**D**_**agTXo1–3**_	AG	OFS	20.0		
**D**_**Ae1–3**_	Q	ethanol		20.0	
**D**_**Qe1–3**_	Q	ethanol			20.0
**D**_**TXASAe1–3a**_	Q	ethanol	20.0	2.5	
**D**_**TXASAe1–3b**_	Q	ethanol	20.0	5.0	
**D**_**TX,ASAe1–3c**_	Q	ethanol	20.0	10.0	
**D**_**TX,QUEe1–3a**_	Q	ethanol	20.0		2.5
**D**_**TX,QUEe1–3b**_	Q	ethanol	20.0		5.0
**D**_**TX,QUEe1–3c**_	Q	ethanol	20.0		10.0
**D**_**TX,ASAo1–3a**_	Q	OFS	20.0	2.5	
**D**_**TX,ASAo1–3b**_	Q	OFS	20.0	5.0	
**D**_**TX,ASAo1–3c**_	Q	OFS	20.0	10.0	
**D**_**TX,QUEo1–3a**_	Q	OFS	20.0		2.5
**D**_**TX,QUEo1–3b**_	Q	OFS	20.0		5.0
**D**_**TX,QUEo1–3c**_	Q	OFS	20.0		10.0
**D**_**TX,ASA,QUEe1–3**_	Q	ethanol	20.0	10.0	10.0
**D**_**TX,ASA,QUEo1–3**_	Q	OFS	20.0	10.0	10.0
**D**_**agTX,ASAe1–3c**_	NG	ethanol	20.0	10.0	
**D**_**ngTX,ASAo1–3c**_	NG	OFS	20.0	10.0	
**D**_**agTX,ASAe1–3c**_	AG	ethanol	20.0	10.0	
**D**_**agTX,ASAo1–3c**_	AG	OFS	20.0	10.0	

a 1–3: All photodegradation experiments were conducted in triplicate, resulting in the generation of three data sets.

b Q = quarts; NG = neutral glass; and AG = amber glass.

### 2.4 Data Handling

Multivariate Curve Resolution (MCR) encompasses a variety of methodologies designed to separate the contributions of chemicals within experimental data results, often represented as a data matrix. These methodologies have been applied to study various multivariate or multicomponent chemical systems. The MCR method decomposes the experimental data matrix (**D**) into a reduced set of contributions from chemical species (in our case, TX and its degradation products), utilizing a bilinear model derived from the multiway mode of Lambert–Beer’s law.^28,29^

1 $$D={CS}^{T}+E$$

where **D** (*n,m*) represents the experimental data matrix, *n* is the number of samples, and *m* is the number of spectral data points. C (*n,k*) contains the concentration evolution of the *k* components, **S** ^**T**^ (*k,m*) denotes the pure spectra of the *k* species, and **E** (*n,m*) signifies the unexplained variance in the model. The number of components involved in matrix **D** (chemical rank) can be estimated by using Principal Component Analysis (PCA) or Singular Value Decomposition (SVD) algorithms. The chemical rank assumes that the species contributing to the measured spectra exhibit singular values larger than other signal contributions, such as experimental or instrumental noise. However, in some instances, rank deficiency may occur, leading PCA to identify a chemical rank lower than the actual value. This limitation can be mitigated by the simultaneous processing of data from various sources through augmentation of the data matrix, either row-wise, column-wise, or by combining rows and columns. Once the number of components is determined, the iterative algorithm of Alternating Least Squares (ALS) utilizes a preliminary estimate of the **S** ^**T**^ or **C** matrices, along with a set of constraints, to optimize the MCR model. Constraints such as non-negativity, unimodality, and concentration closure aid in optimizing the results with chemical significance.

The quality and reliability of multivariate resolution can be assessed using explained variance (%*R* ^2^) and lack of fit (LOF %). These metrics aid in evaluating the dissimilarity between experimental matrix **D** and the data modeled by MCR-ALS processing. The equations for these metrics are as follows.

2 $$Lak\;of\;fit\;\left(\%\right)=100\sqrt{\frac{\sum_{i,j}e_{ij}^{2}}{\sum_{i,j}d_{ij}^{2}}}$$

3 $$R^{2}=100\sqrt{\frac{\sum_{i,j}d_{ij}^{2}-\sum_{i,j}e_{ij}^{2}}{\sum_{i,j}d_{ij}^{2}}}$$

in this study, two variants of MCR-ALS techniques were applied to the data sets. The standard soft MCR-ALS (S-MCR-ALS) was first used for a preliminary assessment of all data matrices to perform a more detailed analysis of the degradation kinetics, the combined hard and soft MCR-ALS (HS-MCR-ALS) method was then applied.^28,29^

### 2.5 Molecular Docking

Molecular docking was performed starting from a crystallographic structure of ERα and ERβ retrieved from the online Protein Data Bank (PDB): 3ERT^30^ for ERα and 2FSZ^31^ for ERβ, both complexed with HTX. ERβ also contains a secondary allosteric binding site occupied by an additional molecule of HTX. The molecular structures of the ligands (HTX, TX, *E*-TX, D1, D2, and D3) were built by using the modeling software Avogadro.^32^ Docking calculations were performed by using AutoDock Vina 1.1.2.^33^ Preliminary conversion of the structures from the PDB format was carried out by using the graphical interface AutoDock Tools 1.5.6.^34^ During the conversion, polar hydrogens were added to the crystallographic receptor structures, whereas apolar hydrogens of the ligands were merged with the carbon atom to which they are attached. Full flexibility was guaranteed for the ligands, resulting in 8 rotatable dihedral angles for TX and *E*-TX and 6 for D1, D2, and D3. A single simulation run was carried out in each case at very high exhaustiveness, 16 times larger than the default value.^22^ The binding mode of the ligands was analyzed through visual inspection, and intermolecular interactions were assessed by using the automated protein–ligand interaction profiler PLIP.^35^ Additionally, the inhibition constant (*K*_i_) was calculated based on the binding energy according to the following equation

4 $$\Delta G=RT\;\ln\left(K_{i}\right)$$

where Δ*G*, *R*, and *T* are related to the binding energy (kcal/mol), gas constant (1.987 cal/K mol), and temperature (310.15 K), respectively.^36,37^

Finally, the ligand efficiency was calculated according to the following equation

5 $$LE=-\Delta G/N_{non-H}$$

where *N*_non‑H_ corresponds to the number of non-hydrogen atoms.^38^

## 3 Results and Discussion

### 3.1 Comprehensive Kinetic Investigation of TX Photodegradation in Solution and Formulation Media

Spectrophotometric data recorded during the degradation experiments were organized into 78 data subsets. These included 9 matrices of size 58 × 181 (**D**_**TXe1–3**_, **D**_**ASAe1–3,**_ and **D**_**QUEe1–3**_) from degradation experiments on ethanol solutions of the standards and 3 subsets (**D**_**TXo1–3**_, 58 × 181) from the degradation of TX solubilized in OFS; 36 data sets were generated from the degradation of binary mixtures (18 for TX + ASA and 18 for TX + QUE) and 6 from the ternary mixture (TX + ASA + QUE), both in ethanol or OFS. In addition, 24 data sets were obtained from the photodegradation of TX in both ethanol and OFS, using two different types of containers: neutral borosilicate glass vials (**D**_**ngTXe1–3**_ and **D**_**ngTXo1–3**_) and amber glass vials (**D**_**agTXe1–3**_ and **D**_**agTXo1–3**_). These tests were designed to simulate realistic packaging conditions and assess the photoprotective effect of the container material under ICH Q1B-compliant irradiation. These new experiments were also conducted in triplicate and were designed to evaluate the photoprotective effects of realistic packaging materials. To further explore photostabilization strategies, the same experimental design was applied to samples of TX formulated with ascorbic acid (ASA) as a chemical stabilizer, at a concentration of 10 mg/L (**D**_**agTX,ASAe1–3c**,_ **D**_**ngTX,ASAo1–3c**,_ **D**_**agTX,ASAe1–3c**_, and **D**_**agTX,ASAo1–3c**_). All data sets were included in the global MCR-ALS analysis. The complete experimental design is summarized in Table 1.

Initially, the photodegradation behavior of TX and the selected compounds was investigated under forced light exposure. The spectral data sets obtained from TX degradation in both ethanol and oral formulation solvent (OFS) were processed using the soft Multivariate Curve Resolution–Alternating Least Squares (S-MCR-ALS) algorithm. To fully exploit the capability of ALS to handle multiple experiments simultaneously, an augmented data matrix was constructed by concatenating six individual data sets along the wavelength dimension (column-wise), resulting in the matrix: **D**_**TXaug**_ = [**D**_**TXe1**_;**D**_**TXe2**_;**D**_**TXe3**_;**D**_**TXo1**_;**D**_**TXo2**_;**D**_**TXo3**_].

Singular Value Decomposition (SVD) of this augmented matrix estimated the presence of at least four distinct species in the photodegradation process in both media. Consequently, it was inferred that three degradation products were formed under the experimental conditions applied.

The MCR-ALS provided an initial estimation of the spectral profiles by selecting the pure variables (PUREST), represented by the S matrix in eq 1, for the species involved in the photodegradation process. During the ALS optimization, constraints were applied to ensure non-negative values and preserve chemical meaning in both the spectral and kinetic profiles. Additionally, assuming that all degradation photoproducts were absorbed within the studied spectral region, a closure constraint was imposed on the concentration (kinetic) profiles to maintain mass balance. The explained variance (R^2^) and lack of fit (LOF %) values indicated excellent agreement between the experimental and modeled data (see Table 2). Figure 1 shows the resolved concentration profiles (**C** matrix) and the corresponding pure spectra (**S** ^**T**^ matrix) for the two test media.

**Table 2:** HS-MCR-ALS Analysis Applied to UV/Vis Data Sets, and the Kinetic Rate Calculated Under Photodegradation Conditions

**data matrix**	**container** ^a^	***k***_**TX**_ **(s** ^**–1**^ **)** ^b^	***t***_**0.33**_ **(s)** ^c^
ethanol	Q	9.593 ± 0.089	423
4 components LOF = 3.95% %*R* ^2^ = 99.61%	NG	1.189 ± 0.025	3409
	AG	0.091 ± 0.002	44670
OFS	Q	20.168 ± 0.004	201
	NG	7.896 ± 0.001	514
	AG	0.392 ± 0.014	10342

**stabilizers added to TX** **(mg/L)**			**TX + ASA**	***t***_**0.33**_ (s)	**TX + QUE**	***t***_**0.33**_ **(s)**	**TX + ASA + QUE**	***t***_**0.33**_ **(s)**
ethanol	2.5	Q	6.012 ± 0.060	674	6.350 ± 0.043	639		
10 components LOF = 5.12% %*R*2 = 99.41%	5.0	Q	5.251 ± 0.094	772	4.533 ± 0.019	895		
	10.0	Q	1.852 ± 0.049	2189	2.168 ± 0.032	1870	2.908 ± 0.022	1394
	10.0	NG	0.172 ± 0.014	23 524				
	10.0	AG	0.010 ± 0.007	410 552				
OFS	2.5	Q	11.855 ± 0.072	342	18.254 ± 0.079	222		
	5.0	Q	9.447 ± 0.045	429	16.471 ± 0.045	246		
	10.0	Q	2.595 ± 0.036	1563	8.139 ± 0.095	498	4.379 ± 0.038	926
	10.0	NG	0.671 ± 0.011	6046				
	10.0	AG	0.022 ± 0.009	184 457				

a Q = quartz; NG = neutral glass; AG = amber glass.

b All kinetic constant values are expressed as being multiplied by 10^–4^.

c *t*_0.33_ = time required for 33% degradation of the initial drug concentration.

**Figure 1:**
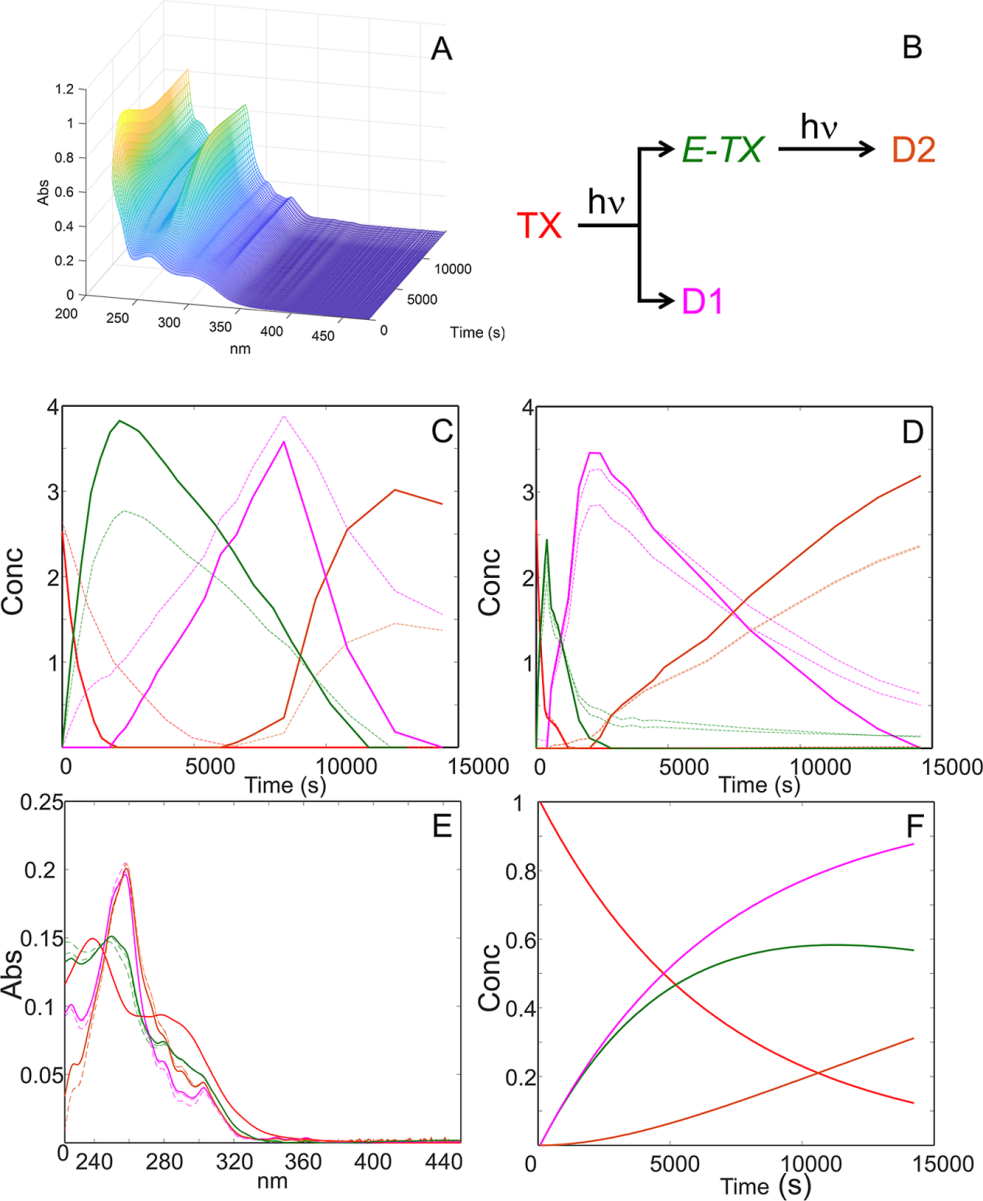
Multivariate resolution of spectral data recorded during photodegradation experiments: (A) 3D plot of the recorded matrix in ethanolic solution; (B) kinetic model of TX photodegradation; (C) concentration profiles for subset **D**_**TXe1–3**_; (D) concentration profiles for subset **D**_**TXo1–3**_; (E) resolved spectral profiles; (F) concentration profiles applying the kinetic constraint; TX (red line), *E*-TX (green line), D1 (pink line), D2 (orange line), and feasible band boundaries (dashed line).

Regarding the kinetic pathway of photodegradation, TX undergoes isomerization and photocyclization reactions in both media. The pathway initially leads to the formation of the *E*-TX isomer. Successively, TX and the newly formed *E*-TX underwent cyclization, resulting in the production of the two phenanthrene derivatives, D1 and D2, respectively.^12^ The initial application of the S-MCR-ALS algorithm revealed rotational ambiguities, especially in the concentration matrix (**C**), and to a lesser extent in the spectral matrix (**S** ^**T**^). This was evident from the analysis of band boundaries using the MCR-BANDS routines (Figure 1) and was likely due to the difficulty in distinguishing overlapping spectral and kinetic contributions from TX, *E*-TX, D1, and D2.^24^

In the case of rotational ambiguities, the adoption of additional constraints in MCR modeling can effectively help in reducing them. Beyond the standard constraints like non-negativity, unimodality, and closure, other powerful tools for minimizing these ambiguities include the use of local rank and/or selective constraints, simultaneous analysis of multiple data sets, and the application of hard modeling routines. The augmented matrix **D**_**TXaug**_ was then reprocessed using HS-MCR-ALS, which employs nonlinear curve-fitting routines (kinetic constraints) to iteratively refine the concentration profiles resolved by MCR-ALS. By considering a specific kinetic model (Figure 1B), this method allowed the optimization of the kinetic rate constants, thereby enhancing the resolution of the concentration profiles of the species formed during the experiments and reducing the rotational ambiguities observed in the S-MCR-ALS results. HS-MCR-ALS results are listed in Table 2 and Figure 1. In the experiments conducted in ethanol (*k*_TX_ = 9.593 ± 0.089), TX exhibited moderate degradation, with a *t*_0.33_ (time required for 33% degradation of the initial drug concentration) of about 7 min, continuing to diminish until it was completely degraded. In contrast, in OFS (*k*_TX_ = 20.168 ± 0.004), the degradation process was markedly faster, with a *t*_0.33_ of 3.5 min, and fully disappeared after 30 min of exposure. The markedly faster photodegradation of tamoxifen observed in the OFS compared to ethanol is likely attributable to the presence of excipients such as polyols (e.g., glycerol, sorbitol, and propylene glycol) and aromatic flavoring agents, which can act as photosensitizers or promote radical-mediated oxidative processes under UV exposure. These excipients have been shown to facilitate the generation of reactive oxygen species (ROS) or undergo light-induced reactions that may catalyze the degradation of coformulated drugs.^39–41^

The MCR-ALS results obtained from the analysis of the time-dependent UV data were confirmed by a chromatographic investigation. HPLC analysis of degraded TX samples revealed three additional peaks, corresponding to *E*-TX (RT 10.25 min), D1 (RT 15.88 min), and D2 (RT 17.10 min), in addition to the TX signal (RT 8.51 min). The UV absorption spectra of these peaks closely matched the pure spectral profiles resolved via MCR-ALS, confirming the identities of the degradation products. Moreover, the chromatographic retention times and spectral characteristics were also compared to those reported in previous literature studies, particularly those involving spectroscopic and LC–MS analyses of tamoxifen photodegradation.^12,13^ This cross-validation among experimental chromatography, multivariate spectral resolution, and established references strengthens the robustness and chemical plausibility of the proposed degradation pathway and supports the correctness of the MCR-derived component profiles.

### 3.2 Stabilization of the Liquid Formulation through the Addition of Chemical Stabilizers

To enhance the photostability of tamoxifen in liquid formulations, we investigated two complementary stabilization strategies: the use of chemical stabilizers and the selection of appropriate packaging materials. For the chemical approach, the effects of two chemical species known for their stabilizing properties, ascorbic acid (ASA) and quercetin (QUE), were studied. Quercetin has been shown to effectively stabilize various compounds against UV-induced degradation, primarily due to its potent antiradical properties and ability to quench reactive oxygen species (ROS) generated during UV exposure.^21,42^ Similarly, ascorbic acid plays a vital role in photoprotection, mitigating oxidative stress in cells exposed to UV radiation and thereby safeguarding cellular integrity.^43,44^

The augmented data matrix was further enriched by including spectral data resulting from the photodegradation of ASA and QUE in ethanolic solution along the column direction. Additional experiments were conducted on binary mixtures, in which each stabilizer, in the range of 2.5–10 mg/L, was individually added to the TX samples, and on ternary mixtures to evaluate potential synergistic effects from the simultaneous use of ASA and QUE. The matrix processing was performed to interpret, within a single computational process, the behavior of the three substances TX, ASA, and QUE when exposed to a light source under stressed conditions. The multivariate modeling successfully identified 10 chemical species within the augmented matrix: 4 related to TX and 3 for each stabilizing agent. The stabilizing effect of ASA and QUE was confirmed and was found to increase with higher concentrations. The results of the chemometric kinetic processing are shown in Table 2. The maximum addition of ASA (10 mg/L) was able to slow the kinetics of TX, achieving a kinetic constant of 1.852 × 10^–4^ in ethanol and 2.595 × 10^–4^ in OFS (green line in Figure 2G), corresponding to a *t*_0.33_ of 14.8 and 11.3 min, respectively. A milder stabilization was observed with the addition of QUE, with *k* values of 2.168 × 10^–4^ (*t*_0.33_ = 10.5 min) and 8.139 × 10^–4^ (*t*_0.33_ = 6.2 min) in ethanol and OFS (red line in Figure 2G), respectively (Figure 2). The experiment conducted to assess the potential combined effect of ASA and QUE on the photostabilization of TX yielded less favorable results (blue line in Figure 2G) compared with the binary combinations. While the presence of both stabilizers resulted in some degree of stabilization, with a reduction in the *k* value for TX degradation, the achieved stabilization was inferior to that observed with ASA alone, which proved to be the most effective stabilizer.

**Figure 2:**
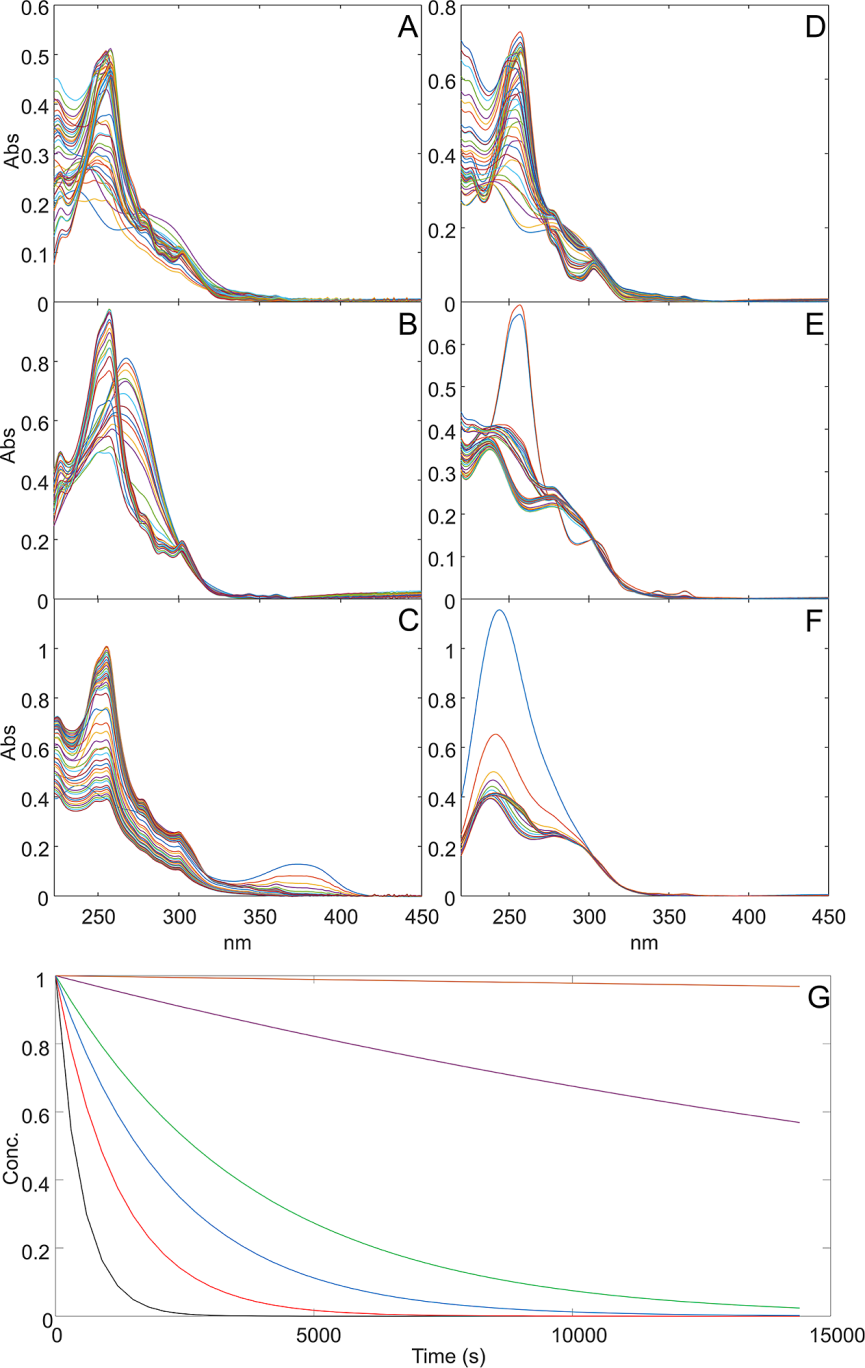
Degradation experiments on binary and ternary mixtures: spectroscopic data recorded for subset **D**_**TXo1–3**_ (A), **D**_**TX,ASAo1–3a**_ (B), **D**_**TX,QUEo1–3c**_ (C), **D**_**ngTXo1–3**_ (D), **D**_**agTXo1–3**_ (E), and **D**_**agTX,ASAo1–3**_ (F); degradation kinetics of TX (G), **D**_**TXo1–3**_ (black line), **D**_**TX,ASAo1–3a**_ (green line), **D**_**TX,QUEo1–3c**_ (red line), **D**_**TX,ASA,QUEo1–3**_ (blue line), **D**_**agTXo1–3**_ (purple line), and **D**_**agTX,ASAo1–3**_ (brown line).

In parallel, we explored the impact of packaging material by comparing degradation in quartz cuvettes (Q), clear borosilicate glass (NG), and amber glass vials (AG). Under identical ICH Q1B light exposure, tamoxifen in amber glass exhibited significantly longer *t*_0.33_ values across all media. In ethanol, TX *t*_0.33_ extended to approximately 1 h in clear glass and to more than 31 days in amber glass, demonstrating a clear protective effect of the packaging material. A similar trend was observed in the more degraded OFS medium, where the *t*_0.33_ rose 21 min in clear glass and reached approximately 3 h in amber glass (purple line in Figure 2G). These findings underscore how both the formulation environment and the type of container play critical roles in modulating the TX photostability. In particular, the use of amber glass, capable of filtering out UV and high-energy visible light, proved to be significantly more effective than clear glass or quartz. When amber glass was combined with 10 mg/L ascorbic acid, the best-performing condition was achieved: TX in OFS showed a t_0.33_ of approximately 114 h (brown line in Figure 2G), representing a more than 7-fold improvement compared to the unstabilized formulation in quartz. This integrated strategy, combining antioxidant excipients with light-protective packaging, offers a practical and highly effective solution to mitigate photodegradation of tamoxifen in liquid pharmaceutical formulations.

### 3.3 Molecular Docking

Cellular responses to estrogen are primarily mediated by two subtypes of the ER: ERα and ERβ. These receptors exhibit variable expression levels and distributions as well as distinct signaling responses. ERα, also known as NR3A1 (Nuclear Receptor subfamily 3, group A), and ERβ (NR3A2) are expressed in various tissues in both male and female bodies. Both receptors play a crucial role in regulating several physiological processes, including cell growth, survival, and differentiation.^45^ The specific role of each receptor subtype during breast cancer development and progression has been clearly established.^7^

In particular, ERα activation generally results in a cell proliferation enhancement, while activation of ERβ can have antiproliferative or antitumor effects. Accordingly, a therapy based on selective ERα antagonists or ERβ agonists could help in limiting tumor growth.^46^ Selective estrogen receptor modulators (SERMs) display a variable agonist/antagonist activity on receptor subtypes, depending on their different tissue expression. For an application in anticancer therapy, antagonist activity in the breast and uterus is required, while agonist activity should be maintained in other tissues, such as cardiovascular, skeletal, and central nervous system, physiologically protected by estrogens.^47^ The structure differences between the two receptor subtypes could account for a diverse biological response to the same compound. Both subtypes consist of 5 domains that share only 57% of the sequence in the ligand binding domain, comprised in AA 302–552 and AA 255–504 for ERα and ERβ, respectively (Figure 3).^48^

**Figure 3:**
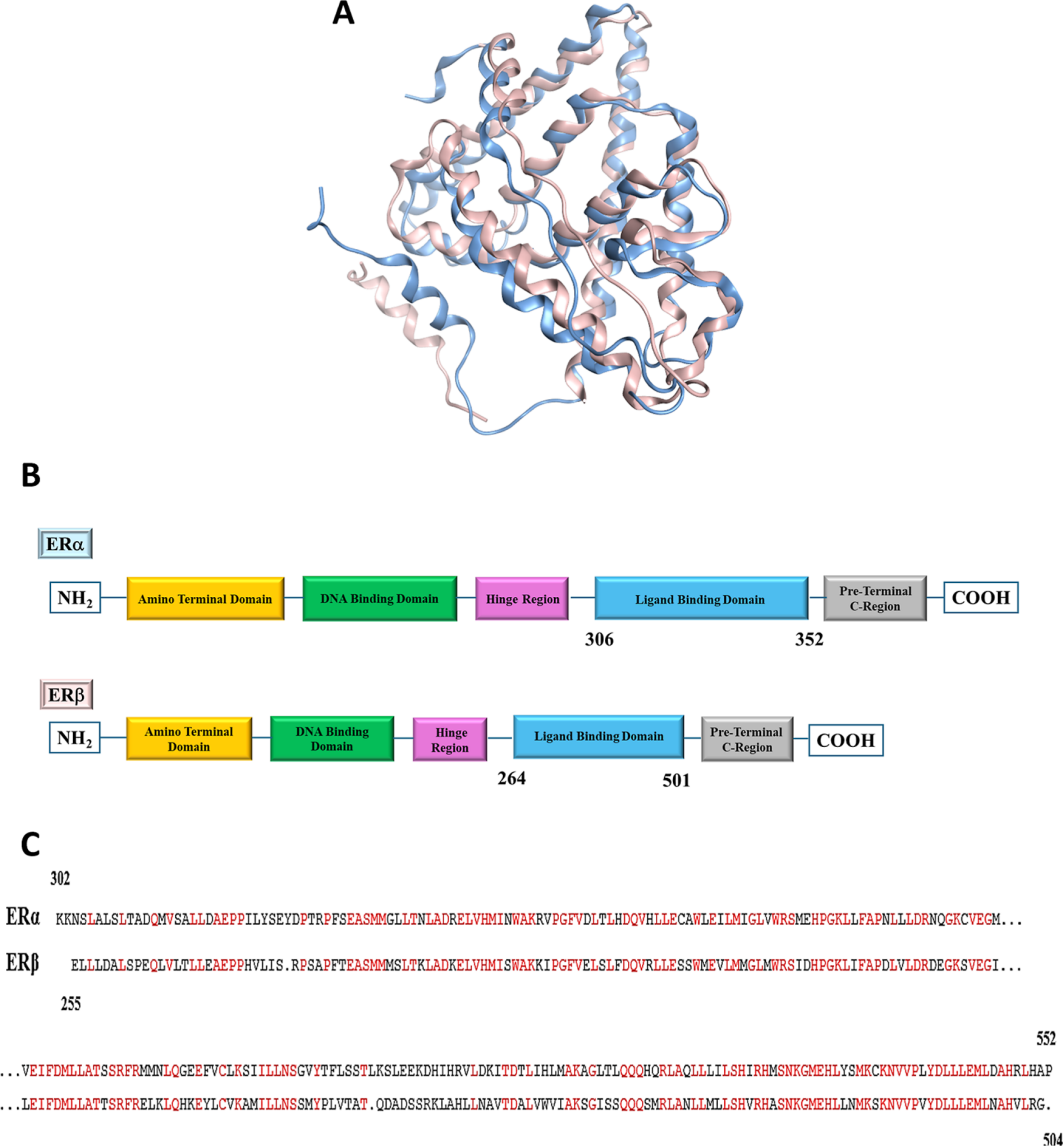
(A) Overlapped representations of ERα (light blue) and ERβ (pink); (B) schematic representation of ERα and Erβ domains; (C) aligned AA sequences for ERα (AA 302–552) and ERβ (AA 255–504), common residues in red.

In light of these findings, a study of molecular docking has been undertaken in order to verify the ability of the four TX photodegradation derivatives to interact with each binding site.

Among the crystallographic structures reported in the Protein Data Bank (PDB), 3ERT^30^ for ERα and 2FSZ for ERβ, in which both receptors are complexed with HTX, have been selected for the present study. 3ERT consists of a single chain of 261 residues, two of which, Asp351 and Arg395, seem necessary for the binding with the ligand. 2FSZ is a homodimer; each monomer, consisting of 246 residues, binds to two molecules of HTX: a first HTX molecule accommodates to the core binding pocket within the ligand-binding domain (site 1), while the second one interacts with an outer region on the surface (site 2). The antagonist activity is mainly associated with the interaction with core site 1, while site 2 allosterically modulates the response.^30,31^ As a first step of our simulation procedure, a redocking experiment has been performed to calculate the binding energy value for each interaction between the crystallographic ligand HTX and the binding sites of both receptors. As a result, a −8.78 kcal/mol value was obtained for ERα, while −8.14 and −5.55 kcal/mol were recorded for ERβ, sites 1 and 2, respectively. The relatively low *K*_i_ values (in the micromolar range) and consistent LE values (∼0.30 kcal/mol per non-hydrogen atom) observed for HTX across all ER binding sites confirm its high binding affinity and efficient interaction with both ERα and ERβ, validating its use as a reference ligand for further docking studies. These values were used as references during successive experiments (Table 3).

**Table 3:** Re-Docking Scores Binding Energy (BE), Inhibition Constant (*K*_i_), Ligand Efficiency (LE) Values, and Key Protein Interacting Residues of HTX within the Binding Sites of ERα and ERβ (Site 1 and Site 2)

	BE kcal/mol	*K*_i_ μM	LE kcal/mol	interactions					
				hydrogen bonds			donor angle°	hydrophobic bonds	salt bridges
				residues	H-A	D–A		residues	
**ERα**	–8.8	0.63	0.30	Glu353	1.61	2.42	142.53	Leu346, Ala350, Trp383, Leu384, Leu387, Phe404, Leu428, Leu525	Asp351
				Arg394	2.09	3.03	157.43		
**ERβ**site 1	–8.1	1.96	0.30	Glu305	2.00	2.67	127.21	Leu298, Leu301, Ala302, Trp335, Leu343, Phe356, Ile373, Ile376, Leu380, Leu476	Asp303
				Arg346	1.92	2.73	138.32		
**ERβ**site 2	–5.5	133	0.19					Leu306, Ile310, Val328, Leu331, Glu332, Trp335	

### 3.4 ERα

TX, *E*-TX, D1, D2, and D3 have been subjected to a site-specific docking simulation within the catalytic site of 3ERT, and it was demonstrated that all the compounds are able to fit similarly to HTX by forming several hydrophobic interactions. All compounds interact with Leu525, Ala350, Leu346 (except for D2), and Trp383 (except for D1). Other residues involved in most cases are Leu384, Leu387, Leu428, and Ile424. Furthermore *E*-TX, D1, and D2, similar to HTX, interact through a salt bridge with Asp351. The absence of hydrogen bonding with Glu353 and Arg394, previously detected for the crystallographic ligand, did not affect the overall stability of the complexes. In fact, the calculated binding energy of TX, *E*-TX, D1, and D3 was comparable to that of HTX, while the sole D2 gave rise to a weaker interaction. Furthermore, all ligands, except D2, which displays both reduced binding affinity (*K*_i_ = 184 μM) and the lowest LE (0.19), exhibit *K*_i_ values below 2 μM and LE values around 0.30 kcal/mol, indicating favorable receptor–ligand interactions and a balanced affinity-to-size efficiency (Figure 4 and Table 4).

**Figure 4:**
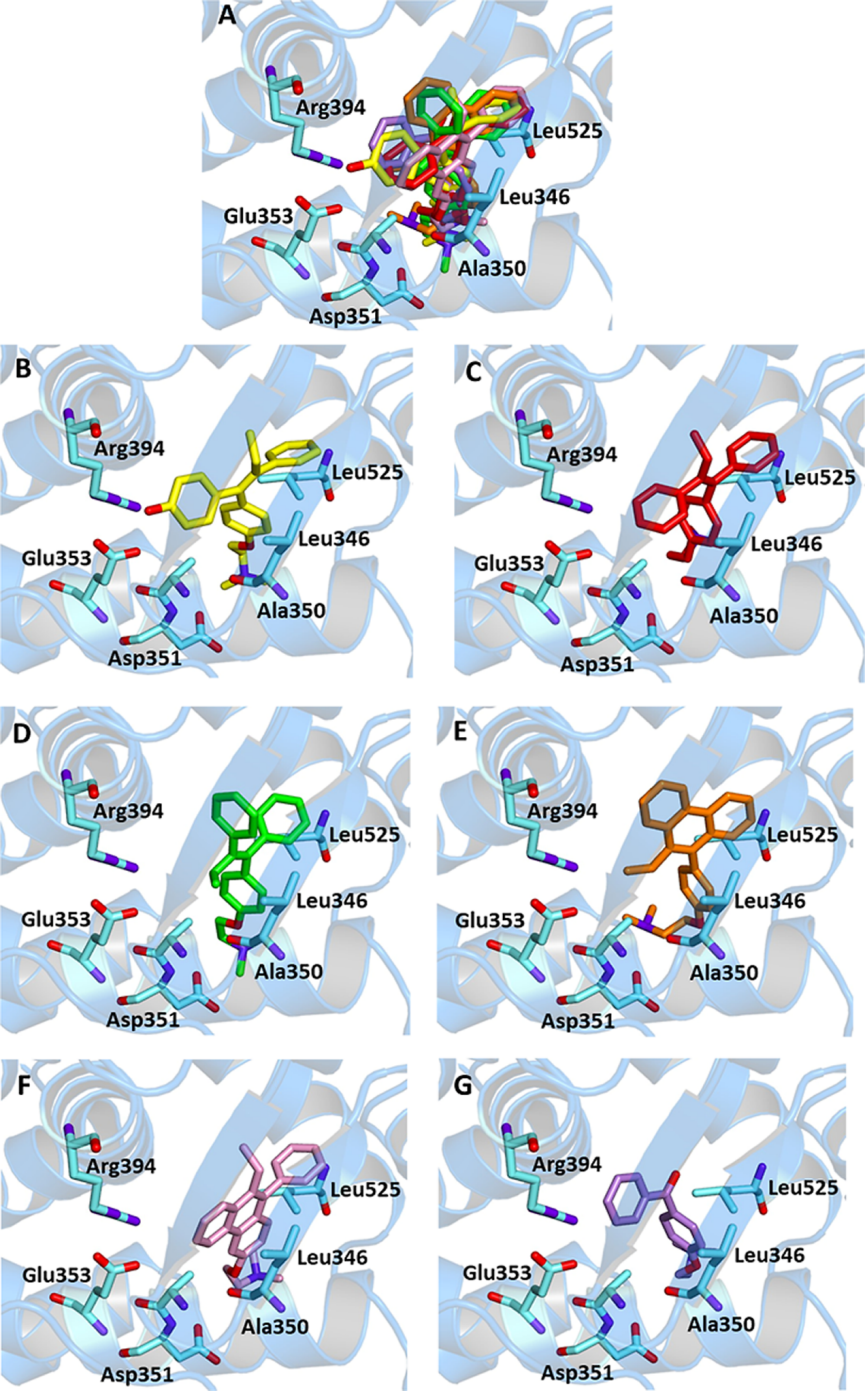
Ligand-binding pocket of the active site of ERα; protein structure is represented in ribbons. The key residues are also indicated. (A) Superimposed binding modes of all the ligands: HTX (yellow), TX (red), *E*-TX (green), D1 (pink), D2 (orange), and D3 (violet); specific binding mode of (B) HTX, (C) TX, (D) *E*-TX, (E) D1, (F) D2, and (G) D3.

**Table 4:** Binding Energy (BE), Inhibition Constant (*K*_i_), Ligand Efficiency (LE), and Key Residues of the ERα Active Site Involved in Ligand Interactions

ligand	BE kcal/mol	*K*_i_ μM	LE kcal/mol	hydrophobic interaction residues	salt bridges
**TX**	–8.9	0.53	0.32	Leu346, Ala350, Trp383, Leu384, Leu387, Ile424, Leu525	
***E*-TX**	–8.3	1.43	0.30	Leu346, Thr347, Ala350, Trp383, Leu384, Leu391, Phe404, Ile424, Leu428, Leu525	Asp351
**D1**	–8.3	1.43	0.30	Leu346, Leu349, Ala350, Phe404, Ile424, Leu428, Leu525	Asp351
**D2**	–5.3	184.00	0.19	Thr347, Ala350, Trp383, Leu384, Leu387, Leu391, Ile424, Leu428, Leu525	Asp351
**D3**	–7.4	6.10	0.37	Leu346, Ala350, Trp383, Leu387,Leu391, Leu525	

### 3.5 ERβ

All ligands were also docked in the core binding pocket of the 2FSZ (site 1). As a result, all compounds were able to enter the binding pocket in a similar manner to HTX, establishing hydrophobic interactions with Ala302, Leu476, Leu298 (with the sole exception of D2), and Phe377 (with the sole exception of D3). Other residues involved are Trp335, Phe356, Ile376, and Leu380. Furthermore, all compounds except for D1 interact through a salt bridge with Asp303. The estimated binding energy was favorable for all compounds, ranging from −7.1 to −9.1 kcal/mol and thus comparable to −9.2 kcal/mol calculated for HTX. All compounds exhibit submicromolar to low micromolar *K*_i_ values and LE values above 0.30, supporting a strong and efficient binding at ERβ site 1. These data underline the conserved inhibitory potential of the photoproducts, especially D1, which closely mirrors the binding efficiency of the reference ligand (Figure 5 and Table 5).

**Figure 5:**
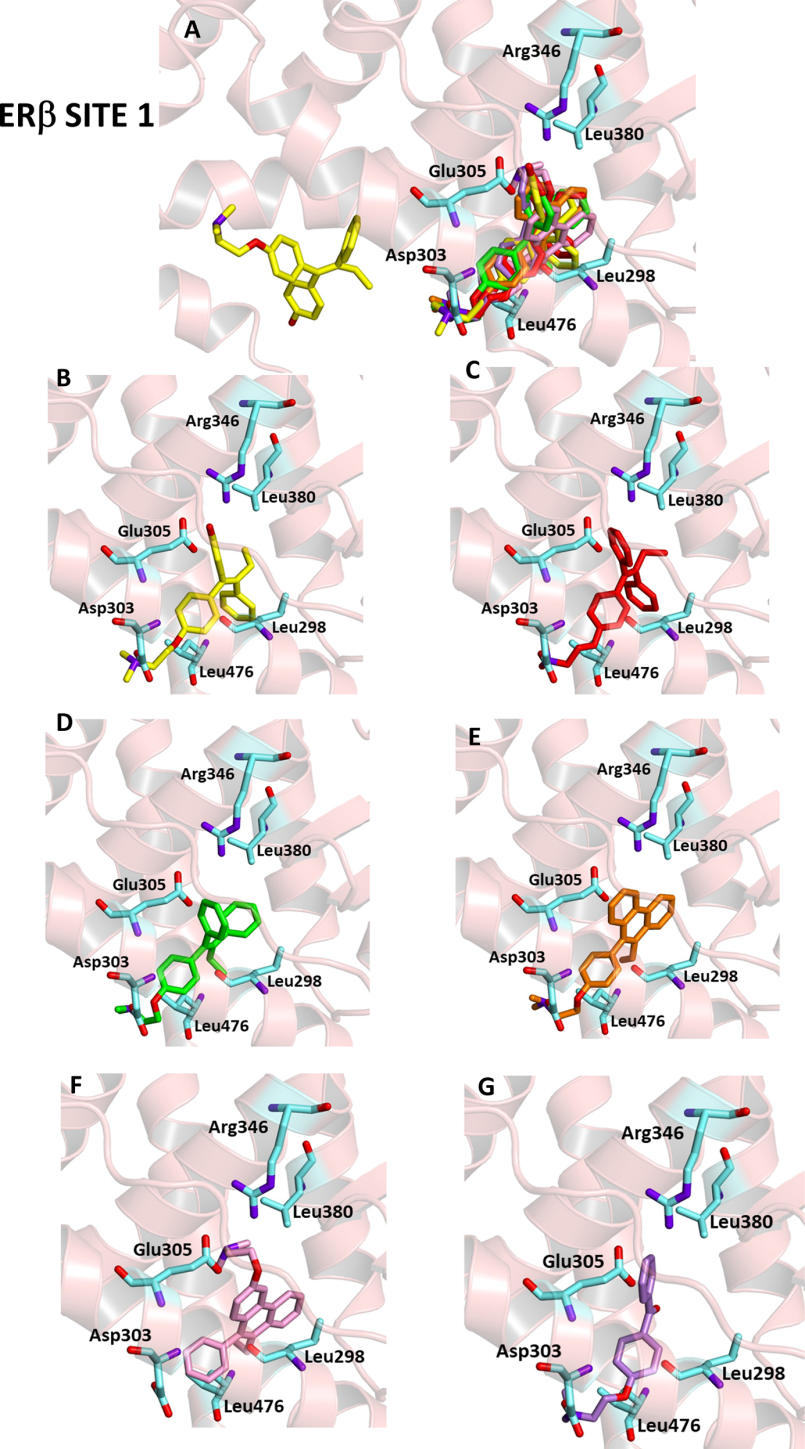
Ligand-binding pocket of the active site of ERβ; protein structure is represented in ribbons. The key residues are also indicated. (A) Superimposed binding modes of all the ligands: HTX (yellow), TX (red), *E*-TX (green), D1 (pink), D2 (orange), and D3 (violet); specific binding mode of (B) HTX, (C) TX, (D) *E*-TX, (E) D1, (F) D2, and (G) D3.

**Table 5:** Binding Energy (BE), Inhibition Constant (*K*_i_), Ligand Efficiency (LE), and Key Residues of the ERβ Active Site Involved in Ligand Interactions^a^

ligand	BE kcal/mol	*K*_i_ μM	LE kcal/mol	hydrophobic interaction residues	salt bridges
**TX**	–9.2	0.33	0.33	Leu298, Ala302, Leu339, Phe356, Ile373, Ile376, Phe377, Leu380	Asp303
***E*-TX**	–8.0	2.30	0.30	Leu298, Leu301, Ala302, Trp335, Phe356, Ile376, Phe377, Leu380, Leu476	Asp303
**D1**	–9.1	0.4	0.32	Leu298, Thr299, Ala302, Trp335, Ile373, Phe377, Leu476	
**D2**	–8.0	2.30	0.30	Ala302, Trp335, Leu339, Phe356, Ile376, Phe377, Leu380, Leu476	Asp303
**D3**	–7.1	9.04	0.35	Leu298, Ala302, Leu339, Leu343, Leu476	Asp303

a Site 1 interacting with the ligands.

In previous studies, the presence of a second pocket region (site 2) on the outer surface of the receptor was identified. This pocket seems to significantly contribute to the overall receptor activity. Thus, the identification of ligands, able to interact also with site 2, would result in a finer modulation of the estrogen activity.^31^ Accordingly, all the compounds studied have been docked into this second pocket region while maintaining occupied site 1 with HTX. In all cases, compounds proved to be able to fit the pocket, even though with binding energy values lower than those observed for site 1. Leu306, Val328, and Leu331 residues are always involved in the formation of hydrophobic interactions, while other hydrophobic interactions or salt bridges contribute to the complex stabilization (Figure 6 and Table 6).

**Figure 6:**
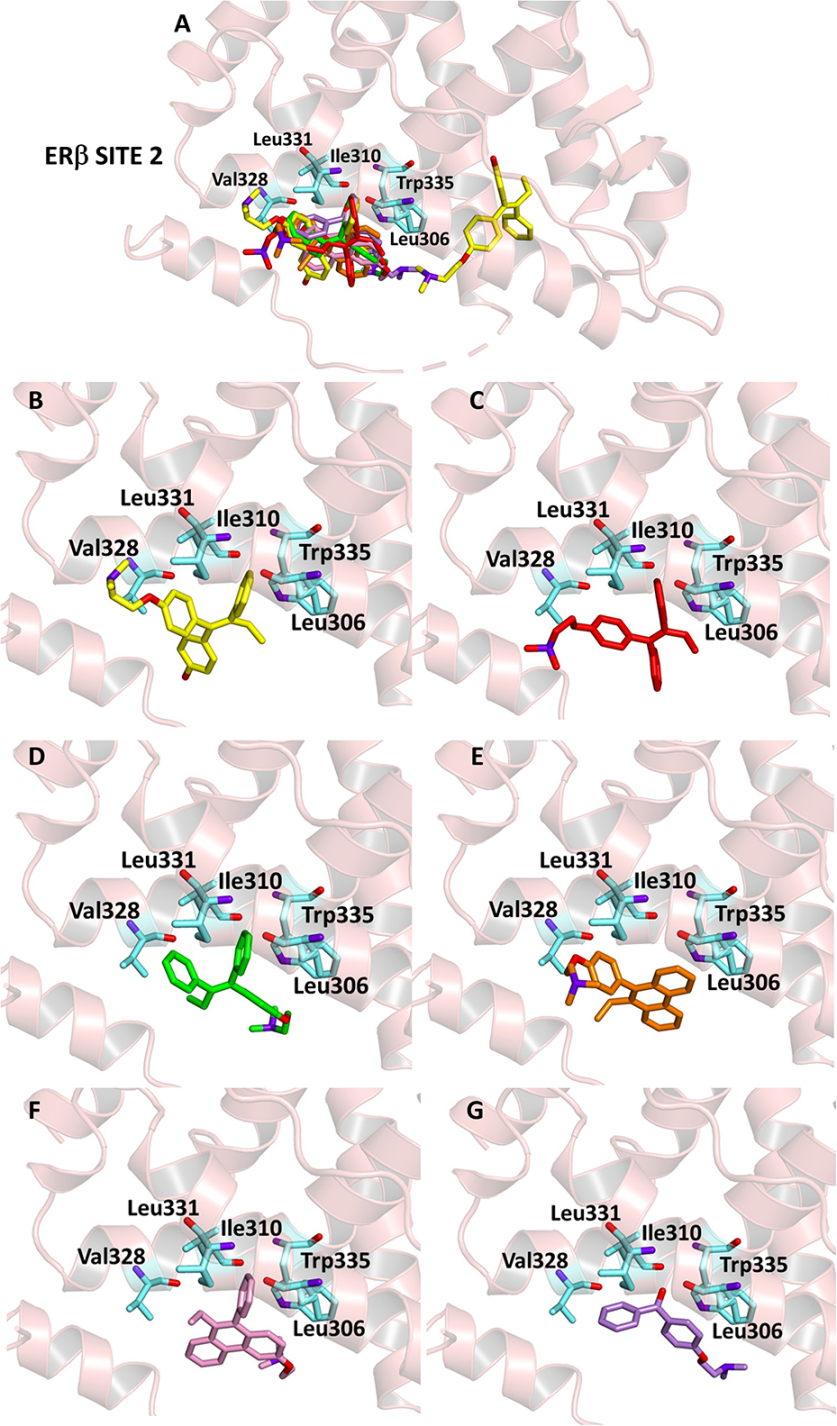
Ligand-binding pocket of site 2 of ERβ when site 1 is occupied by HTX; protein structure is represented in ribbons. The key residues are also indicated. (A) Superimposed binding modes of all the ligands: HTX (yellow), TX (red), *E*-TX (green), D1 (pink), D2 (orange), and D3 (violet); specific binding mode of (B) HTX, (C) TX, (D) *E*-TX, (E) D1, (F) D2, and (G) D3.

**Table 6:** Binding Energy (BE), Inhibition Constant (*K*_i_), Ligand Efficiency (LE), and Key Residues of the ERβ Site 2 Involved in Ligand Interactions^a^

ligand	binding energy kcal/mol	*K*_i_ μM	LE kcal/mol	hydrophobic interaction residues	salt bridges
**TX**	–5.8	81.8	0.21	Leu306, Ile310, Val328, Leu331, Glu332, Trp335	
***E*-TX**	–6.4	30.9	0.23	Leu306, Ile310, Val328, Leu331, Trp335	Glu332
**D1**	–6.8	16.1	0.24	Leu306, Ile310, Val328, Leu331, Glu332, Trp335	Glu332
**D2**	–5.5	133	0.20	Leu306, Val328, Leu331, Glu332, Trp335	Trp335
**D3**	–5.5	133	0.30	Leu306, Ile310, Val328, Leu331	Trp335

a Site 1 interacting with the ligands.

Compared to site 1, the significantly higher *K*_i_ values at ERβ site 2 (ranging from 30.9 to 133 μM) and the lower LE scores reflect a weaker and possibly transient interaction, though still sufficient to suggest a modulatory role for some of the photoproducts, especially D1 and *E*-TX. The overall results obtained proved that all the compounds studied retain the capability to fit both active binding sites of ER, with compound D1 showing the most favorable energy values in all experiments. This compound, directly derived from TX photooxidation, conserves optimal structural features to take into consideration for the development of more specific antiestrogen agents. However, the ability of the photoproducts to bind both ERα and ERβ active sites also raises the hypothesis that they could exert unintended or even toxic biological effects. This consideration strengthens the importance of photoprotection strategies in pharmaceutical formulations, especially in light-sensitive drugs like tamoxifen.

Taken together, the integration of advanced kinetic modeling with molecular docking establishes, for the first time, a direct link between tamoxifen photostability and the pharmacological significance of its degradation products.

## 4 Conclusions

This work provides new insights into tamoxifen photodegradation and highlights its impact on both stability and pharmacological relevance. Degradation studies indicated that TX generates four main derivatives. MCR analysis enabled the identification and quantification of the photogenerated species by the deconvolution of overlapping signals from UV/vis monitoring. Simultaneous chemometric analysis on augmented data matrices allowed us to evaluate the behavior of the drug under all photodegradation conditions. The kinetic modeling revealed that drug degradation occurs significantly faster in the oral formulation than in the alcoholic solution. This accelerated degradation in the oral solution is likely due to the presence of excipients, which may promote radical reactions and enhance the photodecomposition process. Among the tested stabilization approaches, ascorbic acid demonstrated the most effective photoprotective effect, whereas quercetin had a milder impact. The combined use of ASA and QUE did not result in a synergistic improvement, suggesting that their protective mechanisms may not be additive in this system. Further protection was achieved by modifying the packaging environment: amber glass vials dramatically delayed degradation compared with quartz or clear glass. In the best-performing condition, where ASA was combined with amber glass storage, t_0.33_ extended to approximately 114 h, representing a more than 7-fold improvement over the unstabilized system. Molecular docking experiments demonstrated that TX and its degradation compounds can fit into the ERα and ERβ binding site, as well as the accessory binding pocket of ERβ interacting with key amino acid residues. While the preserved binding affinity supports pharmacological relevance, it also raises the possibility that these photoproducts might elicit unintended biological effects. This duality underscores the need for effective photoprotection strategies to minimize potential risks while maintaining drug efficacy. The binding energy, inhibition constants, and ligand efficiency values derived from docking simulations provide compelling evidence that the identified photoproducts, particularly D1, retain pharmacologically relevant affinity toward estrogen receptors, reinforcing their potential contribution to residual biological activity following light exposure. Overall, these results not only expand our understanding of tamoxifen behavior in liquid formulations but also provide a translational framework to improve drug safety, optimize therapeutic efficacy, and support the development of more effective pharmaceutical preparations.

## Supplementary Material



## Data Availability

All original contributions of this study are included in the article. For further information, please contact the corresponding author.

## Notes

Chemical structure of TX, its main active metabolites (HTX and DHTX), and TX degradation products studied (*E*-TX, D1, D2, and D3) (PDF)
