# Supplementary material for: Light-Induced Degradation of Tamoxifen in Liquid Formulations: Multivariate Kinetic Profiling, Stabilization Strategies, and Estrogen Receptor Binding
Source: ACS Omega. 2025 Oct 16;10(42):49569–82. doi: 10.1021/acsomega.5c03822 (PMC12572979; doi:10.1021/acsomega.5c03822)
Supplement: Supplementary file 1 [file ao5c03822_si_001.pdf]

# Light-Induced Degradation of Tamoxifen in Liquid Formulations: Multivariate Kinetic Profiling, Stabilization Strategies, and Estrogen Receptor Binding

Maria Antonietta Occhiuzzi[a], Martina Chieffallo[a], Giuseppina Ioele[a], Giancarlo Di Pinto[b], Giuseppe Cirillo[a], Michele De Luca[a]*, Antonio Garofalo[a] and Fedora Grande[a]

[a] Department of Pharmacy, Health and Nutritional Sciences, University of Calabria, 87036 Rende, Italy

[b] Medical Oncology Unit, Ferrari Hospital, 87012, Castrovillari, Italy

*Email: michele.deluca@unical.it

**Supporting Information**

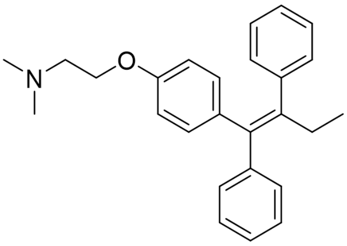

(*Z*)-2-(4-(1,2-diphenylbut-1-en-1-yl) phenoxy)-*N*,*N*-dimethylethanamine

TX

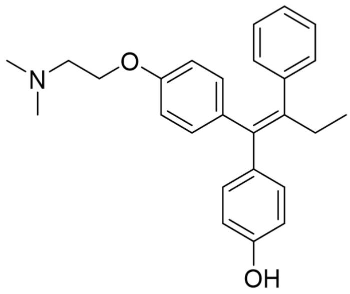

(*Z*)-4-(1-(4-(2-(dimethylamino)ethoxy)phenyl)-2-phenylbut-1-en-1-yl)phenol

HTX

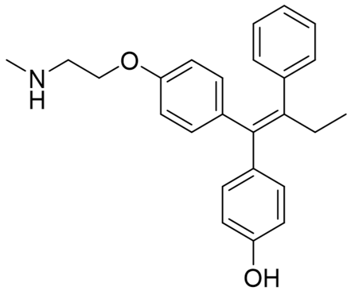

(*Z*)-4-(1-(4-(2-(methylamino)ethoxy)phenyl)-2-phenylbut-1-en-1-yl)phenol

DHTX

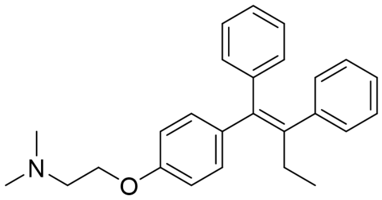

(*E*)-2-(4-(1,2-diphenylbut-1-en-1-yl) phenoxy)-*N*,*N*-dimethylethanamine

*E*-TX

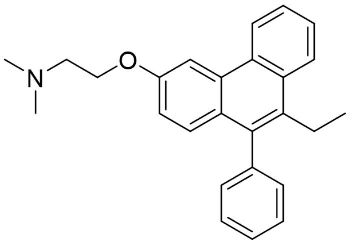

2-((9-ethyl-10-phenylphenanthren-3-yl)oxy) -N,N-dimethylethanamine

D1

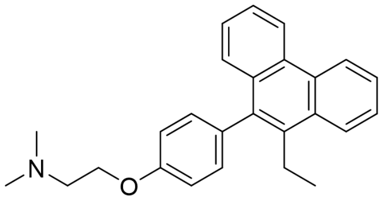

2-(4-(10-ethylphenanthren-9-yl) phenoxy)-N,N-dimethylethanamine

D2

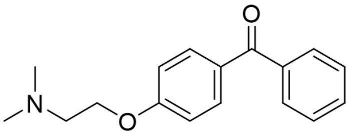

(4-(2-(dimethylamino)ethoxy) phenyl)(phenyl)methanone

D3

**Figure 1S.** Chemical structure of TX, its main active metabolites (HTX and DHTX) and TX degradation products studied (*E-TX*, D1, D2 and D3).
